# Comparison of Three Xylose Pathways in *Pseudomonas putida* KT2440 for the Synthesis of Valuable Products

**DOI:** 10.3389/fbioe.2019.00480

**Published:** 2020-01-17

**Authors:** Isabel Bator, Andreas Wittgens, Frank Rosenau, Till Tiso, Lars M. Blank

**Affiliations:** ^1^iAMB - Institute of Applied Microbiology, ABBt – Aachen Biology and Biotechnology, RWTH Aachen University, Aachen, Germany; ^2^Institute for Pharmaceutical Biotechnology, Ulm-University, Ulm, Germany; ^3^Ulm Center for Peptide Pharmaceuticals, Ulm, Germany; ^4^Max-Planck-Institute for Polymer Research Mainz, Synthesis of Macromolecules, Mainz, Germany

**Keywords:** *Pseudomonas putida*, xylose, metabolic engineering, rhamnolipid, phenazine, pyocyanin, flux balance analysis, heterologous production

## Abstract

*Pseudomonas putida* KT2440 is a well-established chassis in industrial biotechnology. To increase the substrate spectrum, we implemented three alternative xylose utilization pathways, namely the Isomerase, Weimberg, and Dahms pathways. The synthetic operons contain genes from *Escherichia coli* and *Pseudomonas taiwanensis*. For isolating the Dahms pathway in *P. putida* KT2440 two genes (PP_2836 and PP_4283), encoding an endogenous enzyme of the Weimberg pathway and a regulator for glycolaldehyde degradation, were deleted. Before and after adaptive laboratory evolution, these strains were characterized in terms of growth and synthesis of mono-rhamnolipids and pyocyanin. The engineered strain using the Weimberg pathway reached the highest maximal growth rate of 0.30 h^−1^. After adaptive laboratory evolution the lag phase was reduced significantly. The highest titers of 720 mg L^−1^ mono-rhamnolipids and 30 mg L^−1^ pyocyanin were reached by the evolved strain using the Weimberg or an engineered strain using the Isomerase pathway, respectively. The different stoichiometries of the three xylose utilization pathways may allow engineering of tailored chassis for valuable bioproduct synthesis.

## Introduction

For the establishment of a circular bioeconomy, the chemical industry has to overcome the massive and ever-increasing use of fossil resources and the concomitant production of environmental pollutions including greenhouse gases. The alternative is CO_2_ as carbon source, either directly or fixed via chemocatalysis or plants (Olah et al., 2009; Goeppert et al., 2012). CO_2_-fixation though cannot only proceed under natural conditions, there are also many synthetic approaches to convert CO_2_ to valuable products. The conversion of CO_2_ mainly results in the production of biomass in microbes and is well-studied. Hence, it immediately becomes clear that the knowledge about CO_2_-fixation pathways can be utilized to redirect the metabolic flow into the production of chemicals using the synthetic biology arsenal. On the one hand cell-free systems are used to fix CO_2_ via multienzyme cascades (Schwander et al., 2016; Satagopan et al., 2017), on the other hand engineered autotrophic microbes are used for the production of chemicals based on CO_2_ (Lan and Liao, 2011; Angermayr et al., 2012). If the usage of non-autotrophic microbes is desired for the production of multi-carbon compounds via CO_2_-fixation, either existing pathways (e.g., the Calvin cycle) or newly designed pathways have to be introduced into the cells (Guadalupe-Medina et al., 2013; Antonovsky et al., 2016; Bouzon et al., 2017; Schada von Borzyskowski et al., 2018), with the outcome of alternative stoichiometries (Liebal et al., 2018). CO_2_-fixation via plants is a common choice as lignocellulosic biomass is abundant and does not compete immediately with food applications. Lignocellulosic biomass contains about 25–50% hemicellulose (Saha, 2003). In addition to glucose, the pentose xylose is a dominant building block in hemicellulose. For example, bagasse fibers consist of ~23% xylose (Lee, 1997). While wheat straw and corn stover are industrially used as carbon source for bioethanol production (Larsen et al., 2008; Maas et al., 2008; Zhao et al., 2018), exemplifying the feasibility of the alternatives, most other microbes than yeast are still on the experimental scale.

The well-characterized chassis *Pseudomonas putida* KT2440 is known for its versatile metabolism, the richness of cofactors, and high solvent tolerance (Ramos et al., 1995; Nelson et al., 2002; Blank et al., 2010; Tiso et al., 2014). Further, the genome is sequenced and well-annotated (Nelson et al., 2002; Belda et al., 2016; Winsor et al., 2016; Nogales et al., 2019). The structure of the central carbon metabolism and the fluxes on different carbon sources are well-studied for *P. putida* KT2440 (Sudarsan et al., 2014). Further, many tools for genomic engineering are available (Martínez-García and de Lorenzo, 2011; Poblete-Castro et al., 2012; Nikel and de Lorenzo, 2018). Besides, its non-pathogenicity contributes to it being a chassis relevant for production of valuable products (Nelson et al., 2002; Tiso et al., 2014; Loeschcke and Thies, 2015; Wittgens and Rosenau, 2018).

**Figure 1:**
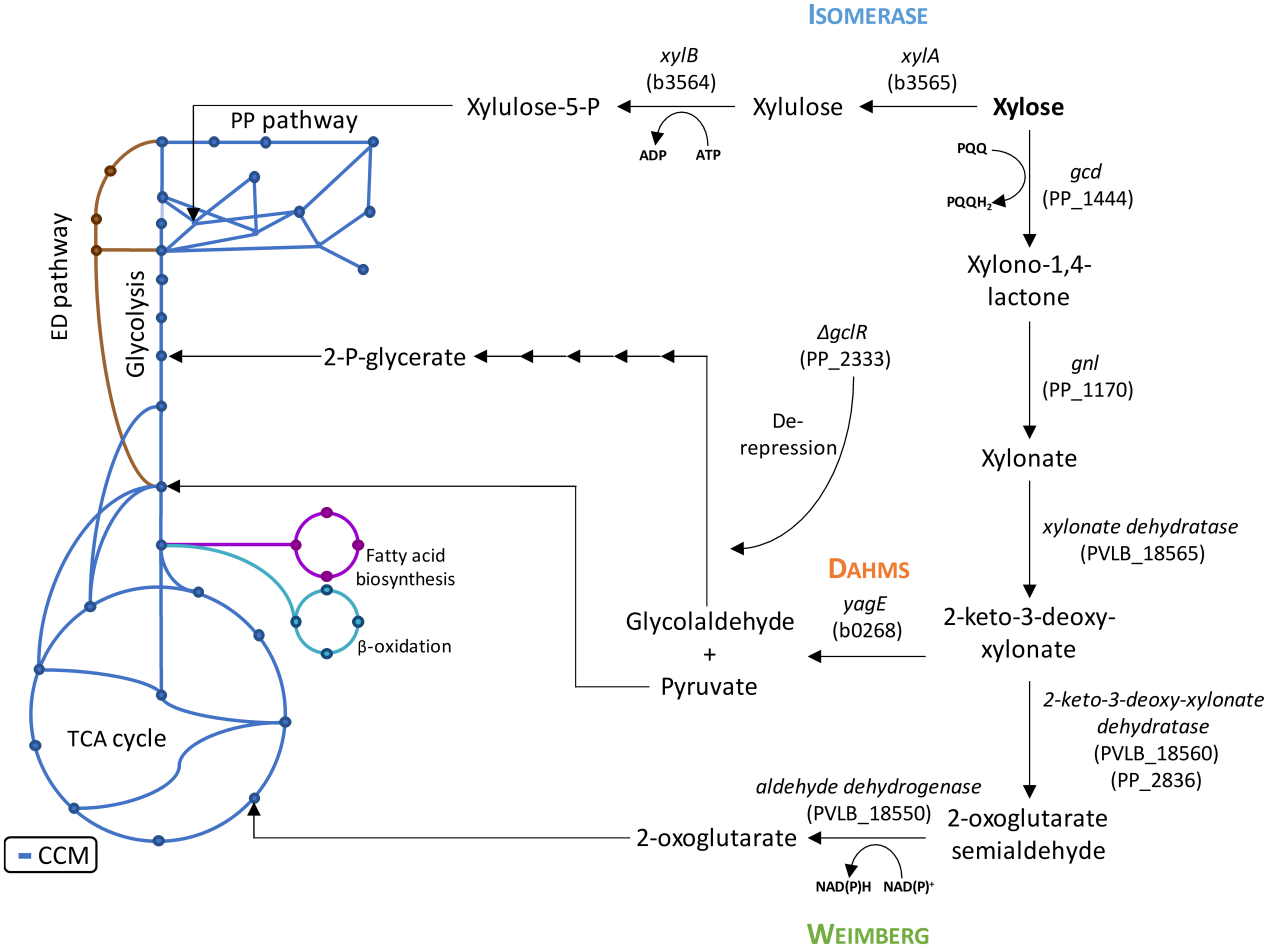
Introduction of xylose metabolism pathways into the central carbon metabolism of *P. putida* KT2440 with the presumed endogenous enzymes and heterologous enzymes from *E. coli* DH5α and *P. taiwanensis* VLB120. The blue lines indicate the central carbon metabolism based on (Sudarsan et al., 2014). The PP numbers represent the locus tag in *P. putida* KT2440, the PVLB numbers represent the locus tag in *P. taiwanensis* VLB120, and the b numbers represent the locus tag in *E. coli* K12, of which *E. coli* DH5α is derived from. ADP, adenosine diphosphate; ATP, adenosine triphosphate; NAD^+^, nicotinamide adenine dinucleotide; NADH, reduced nicotinamide adenine dinucleotide; PQQ, pyrroloquinoline quinone; PQQH_2_, reduced pyrroloquinoline quinone.

However, *P. putida* KT2440 lacks the capability to metabolize xylose. Microbial assimilation of xylose has so far been observed by three different metabolic pathways: An isomerase and two oxidative pathways called Weimberg and Dahms. The Isomerase pathway is characterized by the presence of a xylose isomerase and xylulokinase. The resulting intermediate xylulose is subsequently phosphorylated to xylulose-5-phosphate and introduced into the pentose phosphate pathway. The Isomerase pathway is originally found in prokaryotes, such as *Escherichia coli* and *Bacillus subtilis* (David and Weismeyer, 1970; Wilhelm and Hollenberg, 1985). The oxidative pathways start with the oxidation of xylose to xylonate. This step is often catalyzed by a periplasmic dehydrogenase. A xylonate dehydratase then converts xylonate into 2-keto-3-deoxy-xylonate. The resulting intermediate is dehydrated to α-ketoglutaric semialdehyde in case of the Weimberg pathway. In case of the Dahms pathway, α-ketoglutaric semialdehyde is split by an aldolase into pyruvate and glycolaldehyde. While pyruvate is directly converted to acetyl-CoA in the central carbon metabolism, glycolaldehyde is further metabolized in several steps to 2-phosphoglycerate (Franden et al., 2018). 2-Phosphoglycerate is then converted in glycolysis. In the Weimberg pathway, an additional oxidation step from α-ketoglutaric semialdehyde to 2-oxoglutarate is present. The latter metabolite is converted in the tricarboxylic acid (TCA) cycle (Figure 1). The Weimberg pathway was reported for example to be present in *P. fragi, Haloferax volcanii*, and *Caulobacter crescentus* (Weimberg, 1961; Stephens et al., 2007; Johnsen et al., 2009). The genes of the latter species were already heterologously expressed in different organisms to establish the Weimberg pathway (Meijnen et al., 2009; Radek et al., 2014; Rossoni et al., 2018). Recently, also *P. taiwanensis* VLB120 was found to be a native xylose-consumer using the Weimberg pathway (Köhler et al., 2015). The Dahms pathway was first found to be present in an unclassified *Pseudomonas* strain (Dahms, 1974). Some of the genes of *C. crescentus* were also heterologously expressed to establish the Dahms pathway in *E. coli*, because *E. coli* harbors a gene coding for an aldolase, which catalyzes the last step of the Dahms pathway (Choi et al., 2017; Cabulong et al., 2018).

*P. putida* KT2440 has already been engineered to utilize xylose via the Isomerase pathway (Le Meur et al., 2012; Dvorák and de Lorenzo, 2018). Furthermore, *P. putida* S12 was equipped successfully with xylose metabolism genes to establish the Isomerase and the Weimberg pathway (Meijnen et al., 2008, 2009). In the studies mentioned above, growth via the Isomerase pathway was rather slow. This phenomenon occurred because most of the substrate was oxidized to the dead-end product xylonate. Further, xylose transport was reported to be inefficient in *P. putida* KT2440. By implementing adaptive laboratory evolution (ALE) growth on xylose could be improved (Meijnen et al., 2008). Using a different approach, Dvorák and de Lorenzo additionally inserted the proton-coupled symporter XylE, also achieving improved growth (Dvorák and de Lorenzo, 2018).

So far, metabolic engineering approaches were limited to metabolic alterations in order to synthesize a target compound based on a previously determined substrate. We here propose to exploit the metabolic potential of microbes for the production of chemicals by electing for the substrate of choice the most suited degradation pathway. Pseudomonads are suitable hosts for the heterologous production of several products, e.g., surfactants, aromatics, terpenoids, and phenazines besides others (Wittgens et al., 2011, 2018; Loeschcke and Thies, 2015; Schmitz et al., 2015b; Tiso et al., 2017; Wynands et al., 2018). However, the usage of xylose for the synthesis of valuable chemicals with *P. putida* KT2440 has not been reported so far.

In this work, we aimed at engineering a chassis, which can be used for the production of desired secondary metabolites using xylose. First, we investigated the theoretical potential for the synthesis of valuable products using the different xylose pathways via flux balance analysis (FBA). Second, we implemented the three different xylose pathways into *P. putida* KT2440 and optimized growth by ALE. Third, we used these strains for the synthesis of mono-rhamnolipids and pyocyanin, a derivative of phenazines. We demonstrate efficient growth on xylose via the oxidative pathways. The results show that using xylose pathways having different stoichiometries leads to differences in substrate consumption, growth, and production rates, as well as in biomass and product yields. We propose to use different xylose degradation pathways in dependence of product needs.

## Materials and Methods

### Bacterial Strains, Media, and Growth Conditions

For strain maintenance and cloning experiments, the strains *P. putida* KT2440 (DSM6125, ATCC47054), *E. coli* DH5α (New England Biolabs, Ipswich, MA, USA), and *E. coli* PIR2 (ThermoFisher Scientific, Waltham, MA, USA) were routinely cultivated in LB medium containing 10 g L^−1^ peptone, 5 g L^−1^ yeast extract, and 10 g L^−1^ NaCl. *P. putida* was cultivated at 30°C and *E. coli* at 37°C. If required, 50 μg mL^−1^ kanamycin or 30 μg mL^−1^ gentamycin were added to the medium to avoid loss of plasmid. After mating procedures, *P. putida* strains were selected on cetrimide agar (Sigma-Aldrich, St. Louis, MO, USA). Growth and production experiments were performed using M9 minimal medium with a final composition (per L) of 8.5 g Na_2_HPO_4_x2H_2_O, 3 g KH_2_PO_4_, 0.5 g NaCl, 1 g NH_4_Cl, 2 mM MgSO_4_, 4.87 mg FeSO_4_x7H_2_O, 4.12 mg CaCl_2_x2H_2_O, 1.5 mg MnCl_2_x4H_2_O, 1.87 mg ZnSO_4_x7H_2_O, 0.3 mg H_3_BO_3_, 0.25 mg Na_2_MoO_4_x2H_2_O, 0.15 mg CuCl_2_x2H_2_O, 0.84 mg Na_2_EDTAx2H_2_O (Sambrook and Russell, 2001), and 10 g glucose for pre-cultures or 10 g xylose for main cultures. Growth experiments were performed in 500 mL shake flasks with 10% filling volume at 200 rpm and in 24-deep well-plates (SystemDuetz; Enzyscreen B.V., Heemstede, The Netherlands) with 1 mL filling volume at 300 rpm.

#### ^13^C-Labeling Experiments

Strains for the isotope labeling experiments were grown under the conditions stated above. The medium contained 50% of 1-^13^C labeled xylose (99% purity, Sigma-Aldrich, St. Louis, MO, USA). When the culture reached pseudo-steady state, samples with defined amounts of biomass (0.3 mg) were taken, hydrolyzed, and derivatized according to Schmitz et al. (2015a). The MS data were processed with iMS2Flux (Poskar et al., 2012), which enables the correction of the data for the presence of naturally occurring isotopes and the determination of the fractional labeling of selected amino acids. The fractional labeling indicates the fraction of ^13^C-labeled carbon atoms.

### Plasmid and Strain Construction

The genes for xylose utilization were amplified from genomic DNA of *E. coli* DH5α and *P. taiwanensis* VLB120 (isolated with High Pure PCR Template Preparation Kit, Roche Holding, Basel, Switzerland). Plasmid construction of pBT-Isomerase, pBT-Weimberg, and pBT-Dahms was planned with NEBuilder Assembly online tool and performed with NEBuilder HiFi DNA Assembly (New England Biolabs, Ipswich, MA, USA) (Gibson et al., 2009). The expression vector pBT including the constitutive P_tac_ promoter (Koopman et al., 2010) was linearized with XbaI (New England Biolabs, Ipswich, MA, USA) prior to assembly. DNA fragments for Gibson Assembly were amplified using Q5 High-Fidelity DNA Polymerase (New England Biolabs, Ipswich, MA, USA) according to the manual. All used primers with their nucleotide sequences are listed in the supplemental information (Supplementary Table 1). For pBT-Isomerase, the operon *xylAB* (b3565 and b3564 from *E. coli* DH5α) was amplified using primers IB-27 and IB-28 and cloned into the linearized pBT vector by Gibson Assembly. To generate pBT-Weimberg, an operon from *P. taiwanensis* VLB120 was used. The first two genes (PVLB_18555 and PVLB_18550), originally on antisense strand, were amplified using primers IB-63 and IB-64 and the following two genes (PVLB_18560 and PVLB_18565) were amplified using primers IB-65 and IB-66. Afterwards, the linearized pBT vector and both amplified fragments were assembled in one reaction. After that, pBT-Weimberg was used as template for pBT-Dahms. The backbone including PVLB_18555 and PVLB_18550 was amplified from pBT-Weimberg with primers IB-67 and IB-74. Amplification of gene PVLB_18565 from *P. taiwanensis* VLB120 was performed using primers IB-68 and IB-124 and the gene for the aldolase *yagE* (b0268 from *E. coli* DH5α) was amplified using the primers IB-118 and IB-125 to create a synthetic operon. All fragments were assembled via Gibson reactions. In a final step to obtain pBT-Dahms, the putative dehydratase PVLB_18550 was deleted from the assembled vector. Therefore, the vector was amplified with primers IB-93 and IB-94. Subsequently, the amplified vector was self-ligated using T4 Polynucleotide Kinase, T4 DNA Ligase, and T4 DNA Ligase Reaction Buffer (all New England Biolabs GmbH, Ipswich, MA, USA) according to the manual.

The resulting plasmids, pBT-Isomerase, pBT-Weimberg, and pBT-Dahms, were transferred individually into chemically competent *E. coli* PIR2 cells using heat shock according to Hanahan (1983). Transformants were selected on LB agar with kanamycin. Positive colonies were verified via colony PCR using OneTaq 2x Master Mix with Standard Buffer (New England Biolabs, Ipswich, MA, USA). The efficiency of colony PCR was increased by lysis of cell material with alkaline polyethylene glycol according to Chomczynski and Rymaszewski (2006). Plasmids were validated by Sanger sequencing performed by Eurofins (Brussels, Belgium). Subsequently, plasmids were isolated with Monarch Plasmid Miniprep Kit (New England Biolabs, Ipswich, MA, USA) and transferred via electroporation in competent *P. putida* cells according to Choi et al. (2006). Electroporation was performed using a GenePulser Xcell (Biorad, Hercules, CA, USA) using a cuvette with a 2 mm gap and the settings 2.5 kV, 200 Ω, and 25 μF. Plasmids pBT-Isomerase and pBT-Weimberg were transferred individually in *P. putida* KT2440 and *P. putida* KT2440 Δ*gcd*. Plasmid pBT-Dahms was transferred individually in *P. putida* KT2440, *P. putida* KT2440 ΔPP_2836, and *P. putida* KT2440 Δ*gclR* ΔPP_2836. Harboring of the plasmids was verified via colony PCR using primers IB-5 and IB-35 (as above).

Deletion mutants were obtained using the I-*Sce*I-based system developed by Martínez-García and de Lorenzo (2011). Briefly, 700 bp upstream and downstream flanking regions of the target sites, named TS1 and TS2 regions, were amplified from the genomic DNA of *P. putida* KT2440 and cloned into the non-replicative pEMG vector by Gibson Assembly. The resulting plasmids, pEMG-*gcd* and pEMG-PP_2836, were transferred individually into chemically competent *E. coli* PIR2 cells using heat shock as described above. Afterwards, the plasmids were verified by Sanger sequencing and transferred from the *E. coli* PIR2 strains into the required *Pseudomonas* strains via triparental mating according to Wynands et al. (2018). The I-*Sce*I-encoding plasmid pSW-2 was used and no 3-methylbenzoate for induction of I-*Sce*I expression was needed according to Wynands et al. (2018). Positive colonies with loss of kanamycin resistance were verified for targeted deletion by colony PCR using OneTaq 2x Master Mix with Standard Buffer. To obtain marker-free and pure clones, the recombinant strains were cured of pSW-2 plasmid by re-inoculation in LB medium without gentamycin and verified again by colony PCR. In this study, the gene for glucose dehydrogenase (PP_1444) was deleted in the wild type, resulting in *P. putida* KT2440 Δ*gcd*. The gene for the putative 2-keto-3-deoxy-xylonate dehydratase (PP_2836) was deleted in the wild type and *P. putida* KT2440 Δ*gclR* (Li et al., 2019), resulting in *P. putida* KT2440 ΔPP_2836 and *P. putida* KT2440 Δ*gclR* ΔPP_2836.

To demonstrate the production of secondary metabolites, two target molecules, rhamnolipids and phenazines, were chosen. For the production of mono-rhamnolipids, the mini-Tn7 delivery transposon vector (pBG) developed by Zobel et al. (2015), which inserts a synthesis module in a single genomic locus of the chromosome, was used. Therefore, plasmid pBG14ffg with a stronger constitutive, synthetic promoter than pBG14g (Sebastian Köbbing, RWTH Aachen University, personal communication) was used as backbone and amplified with primers SK06 and SK07. The genes enabling rhamnolipid production *rhlAB* from *P. aeruginosa* were amplified with primers SK08 and SK09 and cloned into the amplified backbone by Gibson Assembly. The resulting mini-Tn7 vector pSK02 was integrated into the genome of xylose consuming strains via homologous recombination according to Zobel et al. (2015). The integration of the rhamnolipid synthesis module into the *att*Tn7 site was verified by colony PCR as described above. Mono-rhamnolipid producing clones were identified using cetrimide-blood agar plates (7.5% (v/v) sheep blood; Fiebig-Naehrstofftechnik, Idstein-Niederauroff, Germany). If rhamnolipid synthesis occurred, a halo around the colony was visible, because of their hemolytic activity. The 10 clones showing the largest halos were selected for experiments in minimal medium with 10 g L^−1^ xylose to quantitatively examine rhamnolipid production. Subsequently, the best three producers of each strain were subjected to replicate experiments and out of these the best was chosen for further characterization and is described in this study.

Phenazine production was achieved by plasmid-based expression. Plasmid pJNN_*phzA1*-*G1*,*M*,*S* (modified from Schmitz et al., 2015b) was transferred into competent xylose consuming strains via electroporation according to Choi et al. (2006). Harboring of the plasmid was confirmed via colony PCR as described above. Based on Schmitz et al. (2015b), five clones were used to inoculate 1 mL minimal medium containing antibiotics and 10 g L^−1^ xylose at a starting OD_600_ of 0.1 and induced with 0.1 mM salicylate to induce phenazine synthesis. If phenazine synthesis occurred, a blue color, which is typical for pyocyanin production, was observed. The best three producers of each strain were subjected to replicate experiments and out of these the best was chosen for further characterization and is described in this study.

All used strains in this study are listed in Table 1.

**Table 1:** Bacterial strains used in this study.

**Strains and plasmids**	**Characteristics**	**References or sources**
***E. coli***		
DH5α	*supE44*, Δ*lacU169* (*Φ80lacZ*ΔM15), *hsdR17* (${\text{r}}_{{\text{K}}^{-}}$ ${\text{m}}_{{\text{K}}^{+}}$), *recA1, endA1, thi-1, gyrA96, relA1*	Hanahan, 1985
DH5αλpir	λ*pir* lysogen of DH5α; host for oriV(R6K) vectors	de Lorenzo and Timmis, 1994
PIR2	F^−^, Δ*lac169, rpoS*(*Am*), *robA1, creC510, hsdR514, endA, recA1 uidA*(Δ*MluI*)::*pir*; host for *oriV*(R6K) vectors	ThermoFisher Scientific
HB101 pRK2013	Sm^R^*, hsdR*-*M*^+^, *proA2, leuB6, thi-1, recA*; harboring plasmid pRK2013	Ditta et al., 1980
DH5α pSW-2	DH5α harboring plasmid pSW-2 encoding I-*Sce*I nuclease, tool for genomic deletion	Martínez-García and de Lorenzo, 2011
PIR2 pEMG-PP_2836	PIR2 harboring plasmid pEMG-PP_2836	This study
PIR2 pEMG-*gcd*	PIR2 harboring plasmid pEMG-*gcd*	This study
DH5αλpir pTnS-1	DH5αλ*pir* harboring plasmid pTnS**-**1	Choi et al., 2005
DH5α pBT	DH5α harboring plasmid pBT; expression vector; containing the constitutive *tac* promoter	Koopman et al., 2010
DH5α pBT-Isomerase	DH5α harboring plasmid pBT; containing the *xylAB* genes from *E. coli* DH5α	This study
DH5α pBT-Weimberg	DH5α harboring plasmid pBT; containing the PVLB18550/18555/18560/18565 genes from *P. taiwanensis* VLB120	This study
DH5α pBT-Dahms	DH5α harboring plasmid pBT; containing the PVLB18555/18565 genes from *P. taiwanensis* VLB120 and *yagE* gene from *E. coli* DH5α	This study
PIR2 pBG14ffg	PIR2 harboring Tn7 delivery vector pBG14ffg; containing BCD2-*msfgfp* fusion	Köbbing et al., in preparation
DH5αλpir pSK02	DH5αλpir harboring Tn7 delivery vector pSK02 for chromosomal integration; containing *rhlAB* genes from *P. aeruginosa* PA01	This study
DH5α pJNN_*phzA1-G1,M,S*	DH5α harboring plasmid pJNN_*phzA1-G1,M,S;* containing *phzA1-G1,M,S* genes from *P. aeruginosa* PA01	Schmitz
***P. taiwanensis***		
VLB120	Wild type	Panke et al., 1998
***P. putida***		
KT2440	Wild type	Bagdasarian et al., 1981
KT2440 Δ*gcd*	Δ*gcd*	This study
KT2440 Δ*gclR*	Δ*gclR*	Li et al., 2019
KT2440 ΔPP_2836	ΔPP_2836	This study
KT2440 Δ*gclR* ΔPP_2836	Δ*gclR*, ΔPP_2836	This study
KT2440 pIso	Wild type harboring plasmid pBT-Isomerase	This study
KT2440 Δ*gcd* pIso	Δ*gcd* harboring plasmid pBT-Isomerase	This study
KT2440 pWeim	Wild type harboring plasmid pBT-Weimberg	This study
KT2440 Δ*gcd* pWeim	Δ*gcd* harboring plasmid pBT-Weimberg	This study
KT2440 pDahms	Wild type harboring plasmid pBT-Dahms	This study
KT2440 ΔPP_2836 pDahms	ΔPP_2836 harboring plasmid pBT-Dahms	This study
KT2440 Δ*gclR* pDahms	Δ*gclR* harboring plasmid pBT-Dahms	This study
KT2440ΔΔ pDahms	Δ*gclR*, ΔPP_2836 harboring plasmid pBT-Dahms	This study
EM42 Δ*gcd* pSEVA2213_*xylABE*	EM42 Δ*gcd* harboring plasmid pSEVA2213_*xylABE*	Dvorák and de Lorenzo, 2018
KT2440 pWeim2	Isolate of *P. putida* KT2440 pWeim after 17th transfer from laboratory evolution on xylose	This study
KT2440ΔΔ pDahms2	Isolate of *P. putida* KT2440ΔΔ pDahms after 26th transfer from laboratory evolution on xylose	This study
KT2440 pIso_RL	*P. putida* KT2440 pIso with *attTn7::Pffg-rhlAB*	This study
KT2440 pIso2_RL	*P. putida* EM42 Δ*gcd* pSEVA2213_*xylABE* with *attTn7::Pffg-rhlAB*	This study
KT2440 pWeim_RL	*P. putida* KT2440 pWeim with *attTn7::Pffg-rhlAB*	This study
KT2440 pWeim2_RL	*P. putida* KT2440 pWeim2 with *attTn7::Pffg-rhlAB*	This study
KT2440ΔΔ pDahms_RL	*P. putida* KT2440ΔΔ pDahms with *attTn7::Pffg-rhlAB*	This study
KT2440ΔΔ pDahms2_RL	*P. putida* KT2440ΔΔ pDahms2 with *attTn7::Pffg-rhlAB*	This study
KT2440 pIso_PZ	*P. putida* KT2440 pIso harboring plasmid pJNN_*phzA1-G1,M,S*	This study
KT2440 pIso2_PZ	*P. putida* EM42 Δ*gcd* pSEVA2213_*xylABE* harboring plasmid pJNN_*phzA1-G1,M,S*	This study
KT2440 pWeim_PZ	*P. putida* KT2440 pWeim harboring plasmid pJNN_*phzA1-G1,M,S*	This study
KT2440 pWeim2_PZ	*P. putida* KT2440 pWeim2 harboring plasmid pJNN_*phzA1-G1,M,S*	This study
KT2440ΔΔ pDahms_PZ	*P. putida* KT2440ΔΔ pDahms harboring plasmid pJNN_*phzA1-G1,M,S*	This study
KT2440ΔΔ pDahms2_PZ	*P. putida* KT2440ΔΔ pDahms2 harboring plasmid pJNN_*phzA1-G1,M,S*	This study

### Adaptive Laboratory Evolution

For adaptation to xylose, the different xylose-consuming strains were grown in M9 minimal medium containing 10 g L^−1^ xylose. OD_600_ was measured daily and the cells were sequentially transferred to fresh medium with a starting OD_600_ of 0.1. The sub-culturing was carried out 30 times for the isomerase-strain, 17 times for the Weimberg-strain, and 26 times for the Dahms-strain. The inhomogeneous culture was streaked out on LB-agar to obtain single isolates, which were subsequently tested for adaption to xylose in a 96-well-plate in a Growth Profiler 960 (Enzyscreen B.V., Heemstede, The Netherlands).

### Analytical Methods

#### Analysis of Bacterial Growth

The optical density at 600 nm (OD_600_) was measured using an Ultrospec 10 cell density meter (Amersham Biosciences, UK). A correlation between OD_600_ and cell dry weight (CDW) was established. An OD_600_ of 1.0 corresponds with a cell dry weight of 369 mg L^−1^.

#### Analysis of Xylose and Xylonate

Xylose and xylonate concentrations in the supernatant were analyzed in a Beckmann Coulter System Gold High Performance Liquid Chromatography (HPLC) (Beckmann Coulter, Brea, CA, USA) with a Metab-AAC 300 × 7.8 mm separation column (particle size: 10 μm, ISERA GmbH, Düren, Germany), a UV detector 166 (Beckmann Coulter, Brea, CA, USA) at 210 nm and a refractory index detector (RI 2300, Knauer GmbH, Berlin, Germany). Elution was performed with 5 mM H_2_SO_4_ at a flow rate of 0.5 ml min^−1^ at 40°C.

#### Analysis of Rhamnolipids

Reversed-phase chromatography was performed for analyzing mono-rhamnolipid concentrations based on a method developed earlier (Behrens et al., 2016; Tiso et al., 2016). For sample preparation, the supernatant was mixed 1:1 with acetonitrile and stored at 4°C overnight. Subsequently, the mixture was centrifuged at 16,500 g for 2 min. All samples were filtered with Phenex RC syringe filters (0.2 μm, Ø 4 mm, Phenomenex, Torrance, USA). The HPLC system Ultimate 3000 with a Corona Veo Charged Aerosol Detector (Thermo Fisher Scientific, Waltham, MA, USA) was used. For separation, a NUCLEODUR C18 Gravity 150 × 4.6 mm column (particle size: 3 mm, Macherey-Nagel GmbH & Co. KG, Düren, Germany) was used. The flow rate was set to 1 ml min^−1^ and the column oven temperature was set to 40°C. Acetonitrile (A) and 0.2% (v/v) formic acid in ultra-pure water (B) were used as running buffers. The method started with a ratio of 70% buffer A: 30% buffer B and a linear gradient was applied to reach a ratio of 80:20% in 8 min. The acetonitrile fraction was increased linearly from 80 to 100% between 9 and 10 min and decreased linearly to 70% between 11 and 12.5 min. The measurement was stopped after 15 min.

#### Analysis of Phenazines

The concentration of phenazines was determined in a spectrophotometer Cary 60 (Agilent, Santa Clara, CA, USA) by analyzing the blue phenazine derivative pyocyanin. Samples were centrifuged at 16,500 g for 2 min. Afterwards, the supernatant was vortexed and pyocyanin was measured at 691 nm. For quantification, Lambert-Beer's law with an extinction coefficient of 4.31 mM^−1^ cm^−1^ for pyocyanin was used (Filloux and Ramos, 2014).

#### Analysis of Proteinogenic Amino Acids

^13^C-labeling pattern of proteinogenic amino acids were determined by gas chromatography coupled with mass spectrometry (GC-MS) as described in Schmitz et al. (2015a). A Trace GC Ultra coupled to an ISQ single quadrupole mass spectrometer with a TriPlus RSH autosampler (all Thermo Fisher Scientific, Waltham, MA, USA) was used. For separation, a TraceGOLD TG-5SilMS capillary column (length: 30 mm, inner diameter: 0.25 mm, film thickness: 0.25 μm, Thermo Fisher Scientific, Waltham, MA, USA) was used. A sample volume of 2 μL was injected at 270°C with a split ratio of 1:50. The flow rate of the carrier gas helium was set to 1 mL min^−1^ and the oven temperature was kept constant for 1 min at 140°C. The temperature was increased with a gradient of 10°C min^−1^ to 310°C and then kept constant for 1 min. The temperature of the transfer line and the ion source were both set to 280°C. Ionization was performed by electron impact ionization at −70 eV. All raw data were analyzed using Xcalibur (Thermo Fisher Scientific, Waltham, MA, USA).

### Flux Balance Analysis

For the prediction of product yields, the genome-scale model of *P. putida* KT2440, *i*JN1411, was used (Nogales et al., 2017). All simulations were carried out in MATLAB (version R2017b, the Mathworks, Inc., Natick, MA, USA) using the COBRA toolbox (Schellenberger et al., 2011), with the linear programming solver of Gurobi^1^. First, the existing model network was extended by the xylose utilization pathways as well as the biosynthesis routes to the desired products, e.g., mono-rhamnolipids, pyocyanin, and ethylene glycol, using reaction information from KEGG (Ogata et al., 1999). Subsequently, the evaluation of the respective product yields for the usage of the three xylose utilization pathways and their combination was performed. Here, the uptake of xylose for all investigated cases was set to 10 mmol ${\text{g}}_{\text{CDW}}^{-1}$ h^−1^ and the uptake of other carbon sources (e.g., glucose) was set to zero. The production of the target molecule was used as objective function in the extended model.

## Results

### Alternative Xylose Utilization Pathways for Maximal Product Yield

With FBA one can compute the maximal product yield in different scenarios (e.g., growth, maintenance coefficients, aeration). Here, the genome-scale model *P. putida* KT2440 *i*JN1411 (Nogales et al., 2017) was modified by introducing the alternative xylose utilization pathways. In addition, various synthesis reactions for valuable products according to Werpy and Petersen (2004) and chemicals we are working on were introduced into *i*JN1411.

The maximal product yields of 12 of the 14 metabolites computed on xylose were produced by the Isomerase pathway (Table 2). The exceptions of maximal yields of metabolites are such products that are directly synthesized from intermediates of the Weimberg and Dahms pathway. For example, the intermediate 2-oxoglutarate of the Weimberg pathway is an interesting metabolite, because it is introduced into the TCA cycle without carbon loss. The direct conversion of 2-oxoglutarate into glutamate (or proline, OH-proline, etc.) would thus benefit from the Weimberg pathway. This was also computed by FBA with a maximal product yield of 1 mmol mmol^−1^ for the Weimberg pathway, and only 0.83 and 0.75 mmol mmol^−1^ for the Isomerase and the Dahms pathway, respectively. The intermediate glycolaldehyde, using the Dahms pathway for xylose degradation, itself is a relevant chemical for industrial applications and a precursor for ethylene glycol synthesis. Ethylene glycol can neither be synthesized via the Isomerase nor the Weimberg pathway, while the maximal product yield of the Dahms pathway is 1 mmol mmol^−1^. Consequently, the Dahms pathway is a favorable pathway to produce glycols out of xylose. In case of the Isomerase pathway, xylulose-5-phosphate is part of the pentose phosphate pathway and the intermediate erythrose-4-phosphate is a starting metabolite for aromatics synthesis via the shikimate pathway. This explains that the Isomerase pathway has the highest yield for the production of the here synthesized aromatic compound pyocyanin (0.27 mmol mmol^−1^). Moreover, the highest maximal mono-rhamnolipid yield is also possible via the Isomerase pathway (0.14 mmol mmol^−1^) and lower yields for the Weimberg and the Dahms pathway (0.08 and 0.12 mmol mmol^−1^) were computed. This is due to CO_2_ production using the TCA cycle for the Weimberg pathway and converting glycolaldehyde in several steps to 2-phosphoglycerate for the Dahms pathway, also releasing one CO_2_. In addition, the maximal product yields for all possible combinations of the xylose pathways (Isomerase + Weimberg, Isomerase + Dahms, Weimberg + Dahms, and Isomerase + Weimberg + Dahms) were computed. In general, it was observed that always one of the pathways in the combinations is favored and is thus exclusively used. Interestingly, and contrary to the above, the combination of the Weimberg and the Dahms pathways resulted in higher maximal product yields for nine of the 14 metabolites (Table 2) compared to the single pathway strains. Thus, in Table 2 only the combined activities of the latter pathways are also listed. In summary, the *in silico* study indicates that the strain equipped with the Isomerase pathway is best-suited for a range of chemicals including the production of the here chosen metabolites mono-rhamnolipids and pyocyanin. The Weimberg and Dahms pathways are favored in niche applications (Table 2), when xylose is the sole carbon and energy source.

**Table 2:** FBA computed maximal product yield of valuable products for three different xylose utilization pathways (Isomerase, Weimberg, and Dahms) and the combination of the Weimberg (W) and the Dahms (D) pathway.

**Products**	**Maximal product yield (mmol_product_/mmol_xylose_)**				
	**Isomerase**	**Weimberg**	**Dahms**	**Weimberg + Dahms**	
Acetoin	**0.83**	0.5	0.75	0.75	100% D
Di-rhamnolipids	**0.12**	0.07	0.1	0.1	19% W, 81% D
Ethylene glycol	0	0	**1**	**1**	100% D
Fumaric acid	**1.67**	1	1.32	1.38	25% W, 75% D
Gluconic acid	**0.83**	0.5	0.64	0.68	27% W, 73% D
Glutamate	0.83	**1**	0.75	**1**	100% W
Glycerol	**0.81**	0.56	0.47	n.d.^a^	–
HAA	**0.17**	0.1	0.15	0.15	6% W, 94% D
Malic acid	**1.67**	1	1.32	1.38	25% W, 75% D
Succinic acid	**1.04**	1	1	1	100% W
Sorbitol	**0.77**	0.5	0.56	0.63	50% W, 50% D
Threonine	**0.83**	0.5	0.75	0.75	100% D
Mono-rhamnolipids	**0.14**	0.08	0.12	0.12	14% W, 86% D
Pyocyanin	**0.27**	0.19	0.17	0.23	63% W, 37% D

a *Due to constraints in the model, the maximal yield for glycerol cannot be computed for the combination of the Weimberg and the Dahms pathway*. *For the combined pathways, also the percentage of the used pathway is indicated*. *The bold values indicate the highest maximal product yield*.

### The Oxidative Pathways Enable Efficient Xylose Utilization

**Figure 2:**
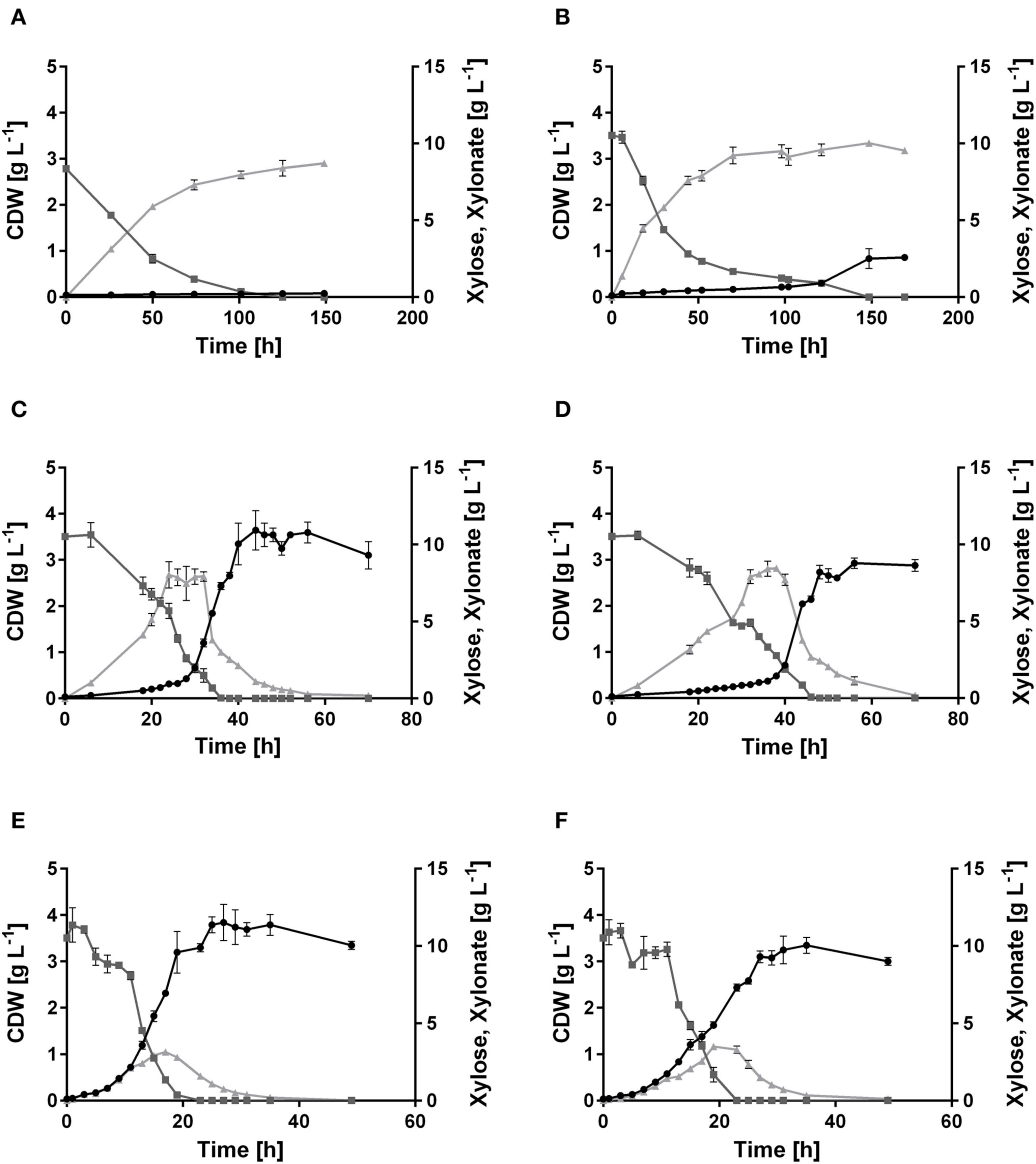
Growth of *P. putida* KT2440, its derivate xylose consuming strains, and the xylose-adapted strains in minimal medium containing 10 g L^−1^ xylose. Consumption of xylose (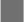, dark gray), formation of xylonate (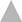, light gray), and cell dry weight (•, black) of **(A)** *P. putida* KT2440, **(B)** *P. putida* KT2440 pIso, **(C)** *P. putida* KT2440 pWeim, **(D)** *P. putida* KT2440ΔΔ pDahms, **(E)** *P. putida* KT2440 pWeim2, and **(F)** *P. putida* KT2440ΔΔ pDahms2. Error bars indicate deviation from the mean (*n* = 3).

A genome analysis using BLAST showed that *P. putida* KT2440 possesses genes (Altschul et al., 1990), which might encode enzymes needed in xylose utilization, but a complete pathway seems to be absent, indeed correlating with no-growth on this sugar (Figure 2A). All genes required for the Isomerase, Weimberg, and Dahms pathways were compared to the genome of *P. putida* KT2440. No homologs for the genes *xylA* and *xylB* from *E. coli* for the Isomerase pathway are present. Additionally, no homologs for the genes PVLB_18565 from *P. taiwanensis* and *yagE* from *E. coli* for the Weimberg pathway and the Dahms pathway, respectively, are present. In contrast, homologous genes of the Weimberg pathway from *P. taiwanensis* are present (homologs to PVLB_18550, PVLB_18555, and PVLB_18560 with sequence identities of 68–72%). HPLC analysis revealed that xylose was consumed by non-growing *P. putida* KT2440, however xylose was converted into the dead-end product xylonate (Figure 2A). Xylonate synthesis proceeds likely via the gene products of *gcd* (PP_1444) and *gnl* (PP_1170) that are besides for glucose also active for xylose and its lactone derivative (Figure 1).

In order to engineer *P. putida* for the utilization of xylose, *P. putida* was equipped with a plasmid for the Isomerase pathway and designated *P. putida* KT2440 pIso. After inoculation, strain *P. putida* KT2440 pIso showed little growth (Figure 2B) with a maximal growth rate of 0.02 h^−1^ and a substrate consumption rate of 0.47 mmol L^−1^ h^−1^ (Table 3). In addition, pBT-Isomerase was introduced into *P. putida* KT2440 Δ*gcd*. This strain is unable to produce the dead-end product xylonate and thus more substrate should be available for biomass formation, as it was reported previously in a study with *P. putida* S12 (Meijnen et al., 2008). The resulting *P. putida* KT2440 Δ*gcd* pIso showed no improved growth compared to *P. putida* KT2440 pIso (data not shown). Nevertheless, these results suggest that the cloned genes *xylAB* from *E. coli* are functionally expressed in *P. putida* KT2440 and allow slow growth of the recombinant strain.

**Table 3:** Growth characteristics of wild type, recombinant, and evolved *P. putida* KT2440 strains on xylose.

**Strain**	**Growth rate μ (h^**−1**^)^a^**	**Lag phase (h)**	**Substrate consumption rate (mmol L^**−1**^ h^**−1**^)^b^**
*P. putida* KT2440	–	–	0.45 ± 0.00^c^
*P. putida* KT2440 pIso	0.02 ± 0.00	100 ± 0	0.47 ± 0.00
*P. putida* KT2440 pWeim	0.30 ± 0.02	24 ± 0	1.94 ± 0.00
*P. putida* KT2440ΔΔ pDahms	0.21 ± 0.02	34 ± 0	1.46 ± 0.00
*P. putida* KT2440 pWeim2	0.21 ± 0.00	3 ± 0	3.05 ± 0.00
*P. putida* KT2440ΔΔ pDahms2	0.21 ± 0.01	3 ± 0	3.05 ± 0.00

a *The growth rate was determined during the exponential phase*.

b *The substrate consumption rate was determined until xylose is depleted. For the wild type, the whole cultivation time was used as reference*.

c *For the wild type, the substrate consumption rate is considered as substrate conversion rate, because it is not able to grow on xylose*.

*Values are the arithmetic mean of biological triplicates. The deviation indicates the standard deviation from the mean (n = 3)*.

In order to establish the oxidative Weimberg pathway in *P. putida* KT2440, the plasmid pBT-Weimberg was transferred into *P. putida* KT2440 resulting in *P. putida* KT2440 pWeim. Interestingly, *P. putida* KT2440 pWeim showed efficient growth with a maximal growth rate of 0.30 h^−1^ and a strong accumulation of xylonate (Figure 2C). This led to a long lag phase of ~24 h (Table 3). We define the lag phase as a phase before growth was observed due to homogenous adaptation. The growth of a small phenotypic subpopulation on the new carbon source due to heterogenous adaptation described by Kotte et al. (2014) is not considered. The observed lag phase indicates a bottleneck in the conversion of xylonate to 2-keto-3-deoxy-xylonate or in the transport from the periplasm to the cytoplasm. Further, the substrate consumption rate was about 4-fold higher (1.94 mmol L^−1^ h^−1^) in comparison to *P. putida* KT2440 pIso (Table 3). These results show that the xylose operon from *P. taiwanensis* is functionally expressed in *P. putida* KT2440 and results in efficient growth. To verify that the glucose dehydrogenase (Gcd) features a side-activity for xylose in *P. putida* KT2440, the plasmid pBT-Weimberg was introduced into *P. putida* KT2440 Δ*gcd* yielding *P. putida* KT2440 Δ*gcd* pWeim. After inoculation, no growth was observed (data not shown). This behavior confirms the assumption that Gcd is active for xylose because xylose needs to be converted to xylonate to establish the Weimberg pathway (Figure 1).

Moreover, the expression of genes encoding the Dahms pathway was targeted. Therefore, the plasmid for the Dahms pathway was introduced into wild type yielding *P. putida* KT2440 pDahms. As described before, genome analysis revealed that a homologous gene of PVLB_18560 is present (PP_2836). By comparison with genes expressing xylose-metabolizing enzymes from *C. crescentus*, PVLB_18560 is assumed to encode a dehydratase, which converts 2-keto-3-deoxy-xylonate to α-ketoglutaric semialdehyde (Figure 1). As a result, it is assumed that the product of the homologous gene PP_2836 in *P. putida* KT2440 is likely to catalyze this reaction. To verify this hypothesis, the plasmid pBT-Dahms was also transferred into *P. putida* KT2440 ΔPP_2836 yielding *P. putida* KT2440 ΔPP_2836 pDahms. *P. putida* KT2440 pDahms reached an OD_600_ of 8.2 after 74 h, while *P. putida* KT2440 ΔPP_2836 pDahms stopped growing after 48 h (final OD_600_ of 2.0). The latter strain showed cell clumping after 74 h, which implies stress. We speculated that low optical density and cell-clumping are caused by an accumulation of the toxic intermediate glycolaldehyde. These findings strongly indicate that PP_2836 encodes a 2-keto-3-deoxy-xylonate dehydratase. In order to overcome the toxic effect of glycolaldehyde, strain *P. putida* KT2440 Δ*gclR* was used. The glyoxylate carboligase oxidase pathway is active in this mutant, thus avoiding glycolaldehyde accumulation (Li et al., 2019). The plasmid pBT-Dahms was introduced into *P. putida* KT2440 Δ*gclR* ΔPP_2836 resulting in *P. putida* KT2440ΔΔ pDahms. In comparison to *P. putida* KT2440 ΔPP_2836 pDahms, strain *P. putida* KT2440ΔΔ pDahms indeed grew to higher cell densities, which led to the conclusion that the genes encoding the enzymes for the Dahms pathway are successfully expressed and the prevention of glycolaldehyde accumulation leads to efficient growth. The growth of *P. putida* KT2440ΔΔ pDahms was further examined and a maximal growth rate of 0.21 h^−1^ and a substrate consumption rate of 1.46 mmol L^−1^ h^−1^ was observed (Table 3). Further, accumulation of xylonate occurred as in the case of the Weimberg strain. Thereby, a lag phase of ~34 h results (Figure 2D).

As stated above, it was observed that not only a long lag phase occurs when the Isomerase pathway is used, but also when the oxidative pathways are used. Regulatory mechanisms are assumed as a cause, but in the case of the oxidative pathways, the used route for metabolization is different. There, the xylose gets oxidized to xylonate via xylono-1,4-lactone by the enzymes encoded by *gcd* and *gnl*. It is assumed that these enzymes are active for xylose, but with a lower specificity and thus, the conversion is slower than for glucose and a long lag phase is the result. Further, accumulation of xylonate was observed, which indicates a bottleneck in xylonate conversion or transport.

### Adaptive Laboratory Evolution Improves Growth on Xylose

**Figure 3:**
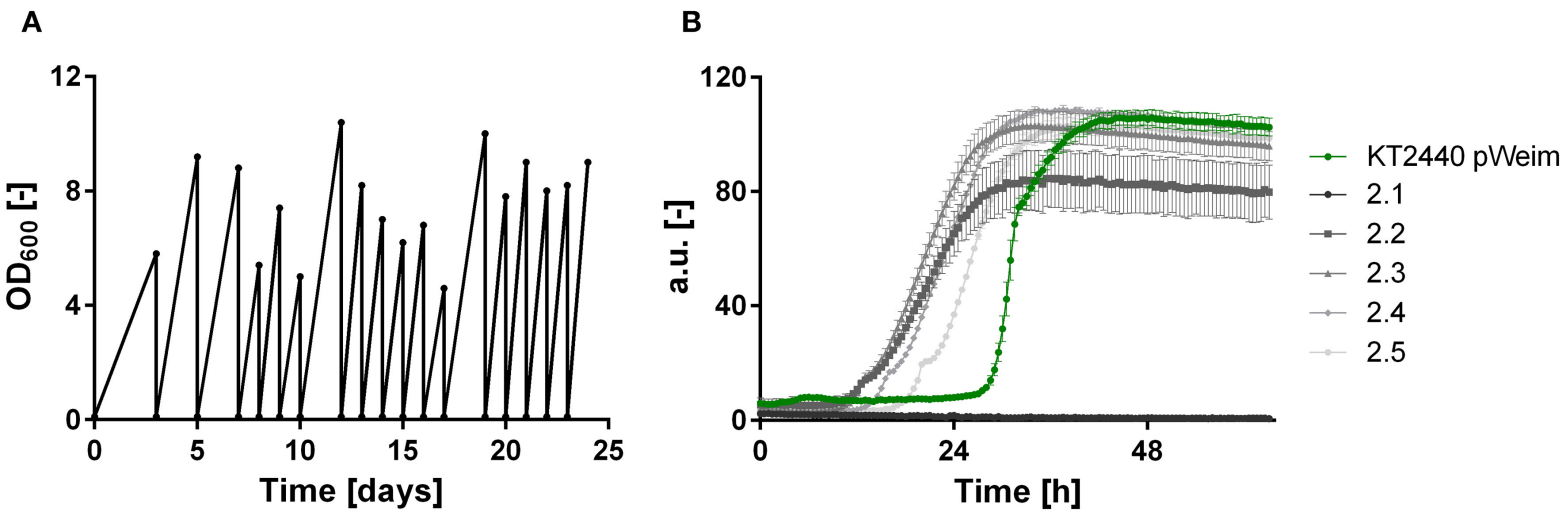
ALE and growth characteristics of isolates of *P. putida* KT2440 pWeim on xylose. **(A)** ALE of *P. putida* KT2440 pWeim on xylose as sole carbon source. A single representative culture is shown, **(B)** Comparison of growth for *P. putida* KT2440 pWeim (•, green) and five isolates from the second ALE culture on xylose. Error bars indicate deviation from the mean (*n* = 3).

It was shown that all xylose metabolism plasmids are functionally expressed resulting in weak growth for the Isomerase pathway and efficient growth for the Weimberg and Dahms pathways, respectively. However, the lag phase of all strains was relatively long. To optimize growth, ALE was applied. For each of the strains, *P. putida* KT2440 pIso, *P. putida* KT2440 pWeim, and *P. putida* KT2440ΔΔ pDahms, two parallel cultures were sequentially transferred to fresh minimal medium with xylose as the sole carbon source (Figure 3A). The performance of *P. putida* KT2440 pWeim and *P. putida* KT2440ΔΔ pDahms was improved after 17 and 26 transfers corresponding to ~115 and 160 generations, respectively. The number of generations was calculated based on the starting OD_600_ and the end OD_600_ of every transfer. Afterwards, 24 isolates were obtained from the culture broth and up to six from each culture were randomly selected and tested for growth on xylose in comparison to the initial strains. In Figure 3B five isolates in comparison to the initial strain *P. putida* KT2440 pWeim are shown. The adaptation toward xylose is not similar for all isolates, as some isolates still had a longer lag phase, suggesting that an isolation step to obtain the best growing strain is required. Hereafter, the best isolates were designated as *P. putida* KT2440 pWeim2 and *P. putida* KT2440ΔΔ pDahms2. The adaptation of *P. putida* KT2440 pIso to xylose was problematic because the strain did not grow anymore after several transfers.

The colonies showing the best performance (*P. putida* KT2440 pWeim2 and *P. putida* KT2440ΔΔ pDahms2) were characterized in detail regarding their growth behavior. Both showed improved growth compared to the initial strains. The lag phase of *P. putida* KT2440 pWeim2 was shortened from ~24 to 3 h (Figure 2E). However, the maximal growth rate of the strain was 0.21 h^−1^. Hence, the lag phase was 1.5-fold reduced compared to the initial strain (Table 3). For *P. putida* KT2440ΔΔ pDahms2, the lag phase was shortened from ~34 to 3 h (Figure 2F). The growth rate remained the same with a value of 0.21 h^−1^ (Table 3). Further, the substrate consumption rate was 3 mmol L^−1^ h^−1^ for both evolved strains, which is about 1.6- and 2-fold higher than the consumption rates of their initial strains.

To identify possible mutations, which are responsible for the reduced lag phase and xylonate accumulation, the plasmids of *P. putida* KT2440 pWeim2 and *P. putida* KT2440ΔΔ pDahms2 were isolated and sequenced. Mutations in the promoter, xylose genes, replication initiator protein, origin of replication, and antibiotic resistance of the plasmids carried by strains *P. putida* KT2440 pWeim2 and *P. putida* KT2440ΔΔ pDahms2 could be excluded. Hence, mutations in coding regions in the genome are expected and will be analyzed in the future.

### The Deletion of PP_2836 Enables the Exclusive Usage of the Dahms Pathway

**Figure 4:**
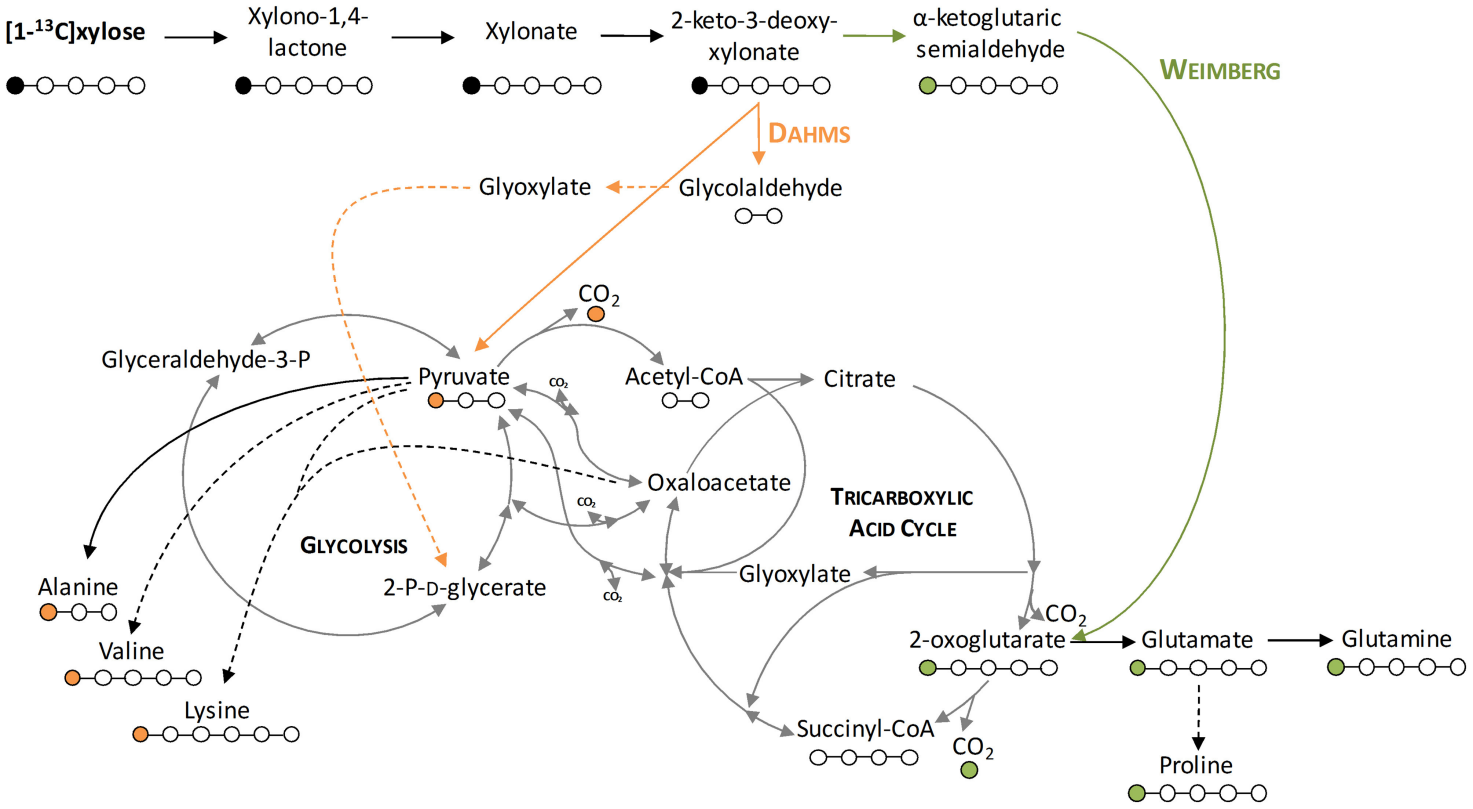
Depiction of the labeling of metabolites resulting from labeled xylose. Shown is the stable isotope introduction into the central carbon metabolism of *P. putida* KT2440. Orange lines indicate the Dahms pathway, green lines indicate the Weimberg pathway, and dashed lines indicate several enzymatic steps. Orange dots indicate the labeling resulting from the Dahms pathway and green dots indicate the labeling resulting from the Weimberg pathway.

To demonstrate that the isolated Weimberg or Dahms pathway are present in the engineered strains, growth experiments with 1-^13^C labeled xylose as substrate were performed. Afterwards, the labeling patterns of the proteinogenic amino acids were analyzed. Since xylose is metabolized via different routes using the Weimberg or the Dahms pathway, the labeling pattern is well-distinguishable (Figure 4). Xylose is converted in several steps to 2-oxoglutarate via the Weimberg pathway, which is a direct precursor of glutamate, glutamine, and proline. This would lead to 1-^13^C labeled glutamate, glutamine and proline. In the central carbon metabolism, 2-oxoglutarate is further processed via the TCA cycle and the labeling is removed via CO_2_ generation. Furthermore, in the case of the Dahms pathway, xylose is converted into pyruvate and glycolaldehyde. Since pyruvate is a precursor for the amino acids alanine, lysine, and valine, they are fractionally labeled. Pyruvate is also converted to acetyl-CoA in the central carbon metabolism, removing the labeled carbon atom by CO_2_ generation.

For these experiments, *P. putida* KT2440 pWeim und *P. putida* KT2440ΔΔ pDahms were used. As seen before, deletion of the gene PP_2836 entails a completely different growth behavior, which indicates that the deletion of PP_2836 is necessary to establish the Dahms pathway. It is assumed that xylose is utilized via both pathways simultaneously (Weimberg and Dahms) in a strain that still harbors PP_2836 and is deficient in *gclR* (to activate degradation of the intermediate glycolaldehyde, see Figure 1). This assumption was experimentally tested by introducing the plasmid pBT-Dahms into *P. putida* Δ*gclR*. The resulting *P. putida* Δ*gclR* pDahms was also used in the labeling experiments.

**Figure 5:**
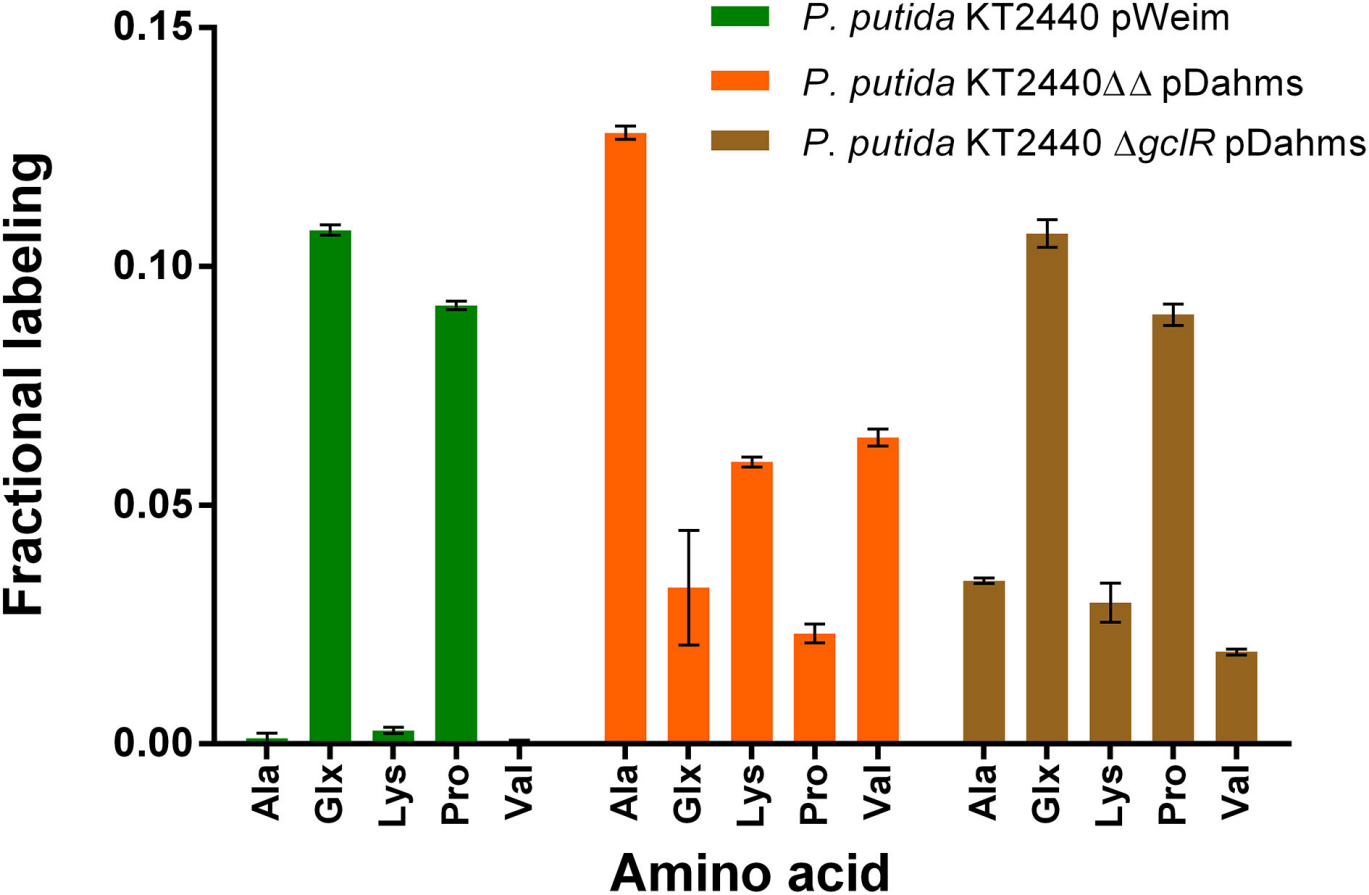
Fractional labeling of amino acids during growth in minimal medium containing 50% 1-^13^C-xylose. Error bars indicate deviation from the mean of three technical replicates from one growth experiment (*n* = 3). Ala, alanine; Glx, glutamate and deaminated glutamine; Lys, lysine; Pro, proline; Val, valine.

Significant distinctions of the fractional labeling with labeled xylose were observed for all three strains (Figure 5). For the Weimberg-strain, alanine, lysine, and valine were almost not labeled at all while glutamate and proline showed a labeling about 0.1. This meets the expectations since 50% of 1-^13^C-labeled xylose was used (one atom out of ten carbon atoms from two xylose molecules should be labeled). For the Dahms-strain, the fractional labeling of the amino acids, which are derived from pyruvate (alanine, lysine, and valine), is about 0.13, 0.06, and 0.07, respectively, and for glutamate and proline below 0.05. Theoretically, a fractional labeling of about 0.17 for alanine, 0.08 for lysine, and 0.1 for valine would be assumed because one out of six, ten, and twelve carbon atoms from two xylose molecules should be labeled, respectively. The observed labeling is a little bit lower than expected, but the distribution meets the expectation since again 50% of 1-^13^C-labeled xylose was used. For more quantitative statements, metabolic flux analysis has to be performed in order to involve fluxes through the central carbon metabolism. Further, as described above the labeling is removed when CO_2_ is generated. However, via anaplerotic reactions the labeled CO_2_ can be reincorporated and ultimately result in labeled glutamate and proline. Nevertheless, both strains showed a distinct labeling pattern, which indicates that the Weimberg and Dahms pathways are truly used in isolation.

Moreover, for *P. putida* KT2440 Δ*gclR* pDahms it was determined that the fractional labeling of glutamate and proline was about 0.1 as in the case of the Weimberg-strain and that the labeling of alanine, lysine, and valine was in between the values of the Weimberg- and the Dahms-strain. These results show that the fractional labeling is a combination of the single fractional labelings of the Weimberg and the Dahms pathway. Thus, it is suggested that the Weimberg and the Dahms pathway are active simultaneously in this strain. Consequently, the above stated declaration that PP_2836 encodes a dehydratase is verified. The deletion of this gene is necessary to obtain a strain, which solely uses the Dahms pathway.

### Variations in Xylose Metabolization Networks Lead to Specialized Microbial Cell Factories

Different stoichiometries of the pathways have an influence on the production yield of specific metabolites. In order to show this dependency, two secondary metabolites (rhamnolipids and phenazines) were chosen for heterologous synthesis in efficient xylose metabolizers. Exemplarily, strains with the individual pathways (Isomerase, Weimberg, and Dahms) were chosen and the production of the initial strains and the adapted strains was investigated. For the production of rhamnolipids, the rhamnolipid synthesis module was integrated as single copy into the genome of all xylose utilizing strains. *P. putida* KT2440 EM42 Δ*gcd* pSEVA2213_*xylABE* (Dvorák and de Lorenzo, 2018) was used as optimized strain using the Isomerase pathway because no evolved *P. putida* strain using the Isomerase pathway was generated. The rhamnolipid producers were designated *P. putida* KT2440 pIso_RL, *P. putida* KT2440 pWeim_RL, *P. putida* KT2440ΔΔ pDahms_RL, *P. putida* KT2440 pIso2_RL, *P. putida* KT2440 pWeim2_RL, and *P. putida* KT2440ΔΔ pDahms2_RL.

**Figure 6:**
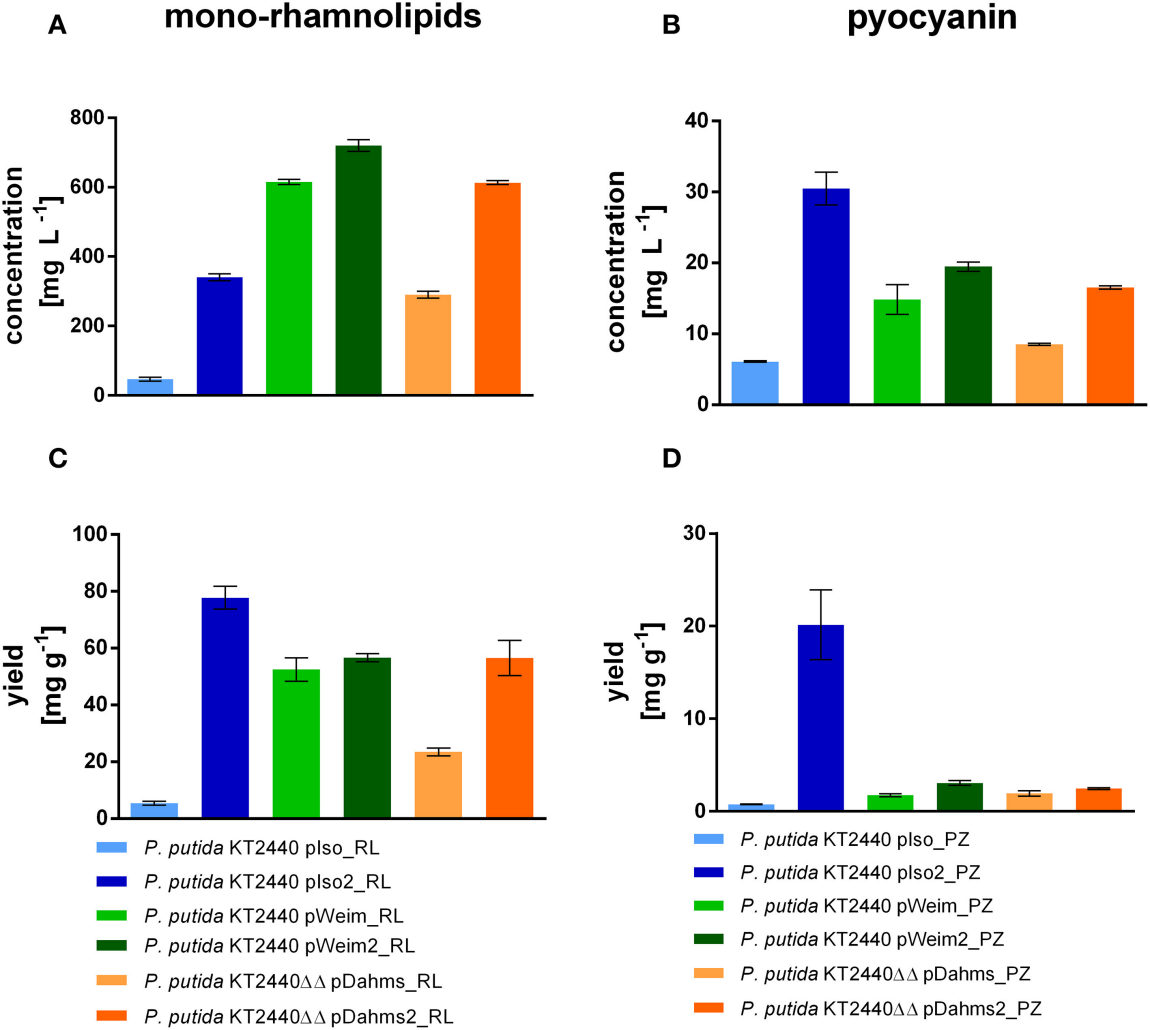
Mono-rhamnolipid and pyocyanin production from xylose by engineered *P. putida* strains. The minimal medium contained 10 g L^−1^ xylose. Shown are the product titers of mono-rhamnolipids **(A)**, the product titers of pyocyanin **(B)**, the yield of mono-rhamnolipids on xylose **(C)**, and the yield of pyocyanin on xylose **(D)**. Error bars indicate deviation from the mean (*n* = 3).

*P. putida* KT2440 pIso_RL, utilizing the Isomerase pathway for xylose degradation, showed only little rhamnolipid production (47 mg L^−1^) after 96 h, because most of the xylose was converted to the dead-end product xylonate. While *P. putida* KT2440 pIso2_RL produced seven times more mono-rhamnolipids after 54 h (Figure 6A), although the cells were clumped and did not reach high optical densities. On the contrary, the strains with the Weimberg and Dahms pathways reached higher rhamnolipid titers. *P. putida* KT2440 pWeim_RL produced 13 times more mono-rhamnolipids than *P. putida* KT2440 pIso_RL in less time (54 h) and the evolved strain with the rhamnolipid production module *P. putida* KT2440 pWeim2_RL produced even more mono-rhamnolipids after 30 h (720 mg L^−1^). Further, *P. putida* KT2440ΔΔ pDahms_RL and *P. putida* KT2440ΔΔ pDahms2_RL produced six times more mono-rhamnolipids after 54 h (290 mg L^−1^) and 13 times more mono-rhamnolipids after 30 h (620 mg L^−1^), respectively. Besides, differences in the substrate consumption were observed. While *P. putida* KT2440 pWeim2_RL and *P. putida* KT2440ΔΔ pDahms_RL consumed all of the provided xylose, xylose and xylonate in the cultures of the other rhamnolipid-producing strains was detected after growth stopped (Supplementary Figure 1). Hence, the ratio of the amount of product synthesized and the amount of substrate consumed (yield) is considered. *P. putida* KT2440 pIso2_RL had the highest yield (78 mg g^−1^), while *P. putida* KT2440 pWeim2_RL and *P. putida* KT2440ΔΔ pDahms2_RL reached comparable yields, however, 1.4-fold lower than *P. putida* KT2440 pIso2_RL (Figure 6C). As expected, the evolved and optimized strains showed a higher rhamnolipid concentration and yield than their initial xylose utilizing strains (Figures 6A,C).

The synthesis of phenazines was achieved by transferring plasmid pJNN_*phzA1-G1,S,M* into all initial xylose utilizing strains and the optimized or evolved strains. The pyocyanin producers were named *P. putida* KT2440 pIso_PZ, *P. putida* KT2440 pWeim_PZ, *P. putida* KT2440ΔΔ pDahms_PZ, *P. putida* KT2440 pIso2_PZ, *P. putida* KT2440 pWeim2_PZ, and *P. putida* KT2440ΔΔ pDahms2_PZ. All strains produced pyocyanin in a similar range (~15–20 mg L^−1^, Figure 6B), but in a different period, except the strains using the Isomerase pathway. *P. putida* KT2440 pIso_PZ produced around three times less (6 mg L^−1^) pyocyanin and *P. putida* KT2440 pIso2_PZ produced roughly two times more (30 mg L^−1^) pyocyanin in comparison to the evolved strain using the Dahms pathway. Pyocyanin was harvested at the same time points as mono-rhamnolipids. Furthermore, as observed in rhamnolipid production experiments, differences in substrate consumption were recognized. *P. putida* KT2440 pIso_PZ converted even more xylose to xylonate than in the rhamnolipid production experiments, which could then not be used for pyocyanin production. Further, less xylose was consumed than in the rhamnolipid production experiment (Supplementary Figure 1). Consequently, the yield was determined and *P. putida* KT2440 pIso2_PZ showed the best yield (20 mg g^−1^) (Figure 6D). Furthermore, the evolved strains reached higher yields than their initial strains (*P. putida* KT2440 pWeim2_PZ: 3.2 mg g^−1^, *P. putida* KT2440ΔΔ pDahms2_PZ: 2.5 mg g^−1^). It was confirmed that the evolved and optimized strains could reach higher product titers and yields than their initial strains, also in case of pyocyanin production (Figures 6B,D). This can be explained by the adaptation toward the novel substrate xylose for *P. putida* KT2440 pWeim2 and *P. putida* KT2440ΔΔ pDahms2. In case of *P. putida* KT2440 pIso2 the production of xylonate is prevented by deletion of the glucose dehydrogenase in comparison to *P. putida* KT2440 pIso and consequently more carbon is available for the production of pyocyanin. Conspicuously, growth was slower for the phenazine-producing strains compared to the rhamnolipid-producing strains and consequently the total consumption of xylose was lower. This might be due to the metabolic burden through the usage of kanamycin to avoid loss of the plasmid harboring the phenazine synthesis genes during cultivation. Further, product toxicity might be a cause for the xylose leftovers. In accordance to the computed data in 3.1., *P. putida* KT2440 pIso2 harboring the rhamnolipid or phenazine genes showed in both scenarios the best yield (Figure 6).

## Discussion

In this study, we integrated three bacterial xylose utilization pathways—Isomerase, Weimberg, and Dahms—in *P. putida* KT2440, to compare *in silico* and *in vivo* the synthesis capacities of these alternative degradation pathways. While other studies focus on heterologous production with *P. putida* KT2440, we aimed at showing the production of various products (i.e., mono-rhamnolipids and pyocyanin) from xylose. Further, we wanted to emphasize that computational analysis can guide strain design, reaching higher yields.

FBA indicates strong preferences for alternative pathways depending on the product of choice due to different stoichiometries. It was determined that mainly the Isomerase pathway is preferred, however the Weimberg and Dahms pathways are favored in niche applications, when for example intermediates of the pathways are precursor for the products. To show this dependency, the xylose pathways and synthesis pathways were integrated in *P. putida* KT2440. In the course of isolating the Dahms pathway, two genes (PP_2836 and PP_4283) were deleted. After successful pathway implementation, the synthesis capacities for mono-rhamnolipids and pyocyanin were investigated. The resulting data matched the computed data. The approach of *in silico* metabolic network design driven by the product of choice, adds another degree of freedom for metabolic engineering.

The computed maximal product yield for both synthesized metabolites, mono-rhamnolipids and pyocyanin, was produced by the Isomerase pathway. In both cases, this was confirmed *in vivo* (Figure 6). While the highest rhamnolipid titer (700 mg L^−1^) was reached by the evolved strain using the Weimberg pathway, the highest yield (78 mg g^−1^) was reached by the strain using the Isomerase pathway. The highest pyocyanin titer (30 mg L^−1^) and yield (20 mg g^−1^) were also reached by the strain using the Isomerase pathway. Taken together, it was shown that the reached yields are dependent on the metabolization route and hence the stoichiometries of the pathway, which can be determined *in silico*.

Many studies deal with the heterologous production by *P. putida* KT2440. Production of biosurfactants, such as hydroxyalkanoyloxy alkanoates (HAA), mono-rhamnolipids, and di-rhamnolipids (Tiso et al., 2017; Wittgens et al., 2017, 2018), terpenoids (zeaxanthin and β-carotene) (Beuttler et al., 2011; Loeschcke et al., 2013), amino acid-derived compounds (e.g., phenazines) (Schmitz et al., 2015b), polyketides/non-ribosomal peptides (e.g., flaviolin, prodigiosin) (Gross et al., 2006; Loeschcke et al., 2013; Domröse et al., 2015), and *N*-methylglutamate (Mindt et al., 2018) was demonstrated. These examples show how diverse the production spectrum in *P. putida* KT2440 can be. To create sustainable production processes in times of high environmental pollution, one searches for alternative substrates (Vanholme et al., 2013). The concept of using xylose or other C_5_ sugars for platform chemicals was already considered before (Werpy and Petersen, 2004). For example, HAA and terpenoids are benefitting from the Isomerase pathway as acetyl-CoA is the precursor (Table 2). Prodigiosin synthesis involves the precursors pyruvate, proline, and malonyl-CoA (Williamson et al., 2006), favoring a combination of the Isomerase and the Weimberg pathway. The Weimberg pathway would be beneficial for synthesis of *N*-methylglutamate. The attempt of using xylose as renewable source for the production of metabolites was already considered in several studies, where *P. taiwanensis* VLB120 was used for the synthesis of mono-rhamnolipids, phenol, and 4-hydroxybenzoate (Tiso et al., 2017; Wynands et al., 2018; Lenzen et al., 2019). *P. taiwanensis* VLB120 utilizes xylose via the Weimberg pathway natively (Köhler et al., 2015), but engineering of the strain and enabling xylose utilization via the Isomerase pathway could enhance product yield on substrate due to the superior stoichiometry.

While the integration of the xylose pathways worked *in vivo*, we observed strong differences between the oxidative and the Isomerase pathway operations. Pseudomonades using the three xylose pathways natively were reported (Hochster, 1955; Dahms, 1974; Köhler et al., 2015), indicating that these pathways are compatible with the Pseudomonades metabolic network. Notably, we did not find any report suggesting the presence of two of the xylose pathways. The implementation of the Isomerase pathway in *P. putida* was shown before (Meijnen et al., 2008; Le Meur et al., 2012; Dvorák and de Lorenzo, 2018; Wang et al., 2019). In accordance to our results, only weak growth was observed in two studies (Meijnen et al., 2008; Dvorák and de Lorenzo, 2018), which was improved by rational or non-rational engineering. In another study, growth on xylose via the Weimberg pathway in *P. putida* was shown to be immediately efficient (Meijnen et al., 2009), matching our results. As already considered by Wang et al. (2019), xylose metabolization seems to be metabolically demanding for *P. putida* KT2440 using the isomerase route and might be the reason for the discrepancies. But how can this difference between the Isomerase pathway and the oxidative pathways be explained? The consideration of other carbon sources indicates that the usage of oxidative pathways is likely to have an advantage for *Pseudomonas* species. The metabolization of the C_5_ sugar arabinose proceeds in *E. coli* via the Isomerase pathway (Laikova, 2001), whereas in other *Pseudomonas* species the utilization of arabinose happens via oxidative steps to form the intermediate pentonic acid (Lockwood and Nelson, 1946; Weimberg and Doudoroff, 1955). In the case of galacturonic acid the utilization starts with an isomerase reaction in *E. coli* (Ashwell et al., 1960). Whereas, in *Pseudomonas* species the first enzyme is a dehydrogenase, which catalyzes an oxidation reaction (Kilgore and Starr, 1959; Richard and Hilditch, 2009). There might be two explanations why *Pseudomonas* favors oxidative pathways. First, the conversion of the substrate into an intermediate acid prevents other microbes from using this substrate, which is an advantage in terms of survival. Consequently, the environment is acidified due to the resulting intermediate acid, which creates a further advantage for propagation. *P. putida* KT2440 is a soil bacterium and is able to cope with extreme conditions, such as nutrient limitation, temperature shifts, and pH changes, in contrast to enterobacteria that occur in nutrient-rich niches (Martins Dos Santos et al., 2004; Reva et al., 2006). Second, the energy metabolism is more flexible, because the dehydrogenases have different redox cofactors. This can be explained using glucose as an example. In general, the redox metabolism is balanced and therefore, the rates of reduction and oxidation of the redox cofactors have to be highly similar (Blank et al., 2010). The electrons, which are released during oxidation of glucose and gluconate, are used to reduce PQQ and FAD^+^ and are feeding directly the electron transport chain. There, PQQ is directly reoxidized by transferring the electrons to ubiquinone in the inner membrane (Ebert et al., 2011; Tiso et al., 2014). The transport of glucose over the membrane costs two ATP per molecule, while only one proton and one sodium ion are necessary for the transport of gluconate and ketogluconate, respectively. Therefore, *P. putida* KT2440 saves energy using the oxidative pathway and does not require additional cofactor regeneration systems.

However, a prolonged lag phase was observed for all three engineered strains. *P. putida* KT2440 pIso had the longest lag phase with 100 h, *P. putida* KT2440 pWeim had a lag phase of 24 h, and *P. putida* KT2440ΔΔ pDahms had a lag phase of 34 h (Table 3). To identify possible bottlenecks, the plasmids were sequenced after ALE. Interestingly, no mutations in the xylose utilization genes and other encoding areas of the vectors could be detected. Different studies showed that mutations in the replication initiation protein or in the antibiotic resistance cassette could enhance growth by lowering the plasmid copy number, and subsequently reducing the need of resources for the synthesis of kanamycin resistance (Jakob et al., 2013; Mi et al., 2016). A second possible bottleneck could be the transport of xylonate from the periplasm to the cytoplasm. *P. putida* KT2440 harbors the transporter GntT (PP_3417), enabling the transport of gluconate from the periplasm into the cytoplasm (Porco et al., 1997). Such a transporter is characterized for *E. coli*. A conceivable possibility would be, that this transporter might be active for xylonate, but due to its high-affinity toward gluconate it might operate slower for xylonate. Another option could be that a slow, low-affinity transporter is used instead. To improve the growth performance and reduce the lag phase, ALE was implemented. Interestingly, the lag phase was reduced significantly, while the growth rates did not increase and in one case even decreased during ALE. This can be explained by positive selection for a reduced lag phase and negative selection for the growth rate. The fast-adapting cells does not have stringently the highest growth rate. After reduction of the lag phase, it can be selected for increased growth rates now. While laboratory evolution is an easy method to increase the overall fitness of a population in laboratory scale, the design of the ALE approach for adaptation of the population is not trivial. Conditions such as time point of transfer, passage size, and growth phase can alter in ALE studies (Charusanti et al., 2010; LaCroix et al., 2015). Batch cultivation and continuous (chemostat) cultivation are the mostly used ALE techniques (Dragosits and Mattanovich, 2013; Gresham and Dunham, 2014; LaCroix et al., 2017). Of these, regularly transferred batch cultures are more popular because effort and costs are rather low. However, this method has several limitations due to varying conditions (LaCroix et al., 2017). Further, this method is slower than an automated ALE because the transfer is usually done on a daily basis. While in automated ALE processes, several parameters, including the optical density and growth rate, are monitored online. If the growth rate increases over the course of the ALE, the passage frequency can automatically be increased. Additionally, cultures probably reach the stationary phase in batch cultivations, which then results in an improved survival in stationary phase or decreased lag phase (Wiser and Lenski, 2015). Thus, it is not surprising that the batch cultivations for adaptation used in this study resulted in a shortage of the lag phase.

The here presented alternative pathways for xylose utilization open another degree of freedom in the design and metabolic engineering of the production strain. Dependent on the product of interest, the experimenter can compute the best network design considering three different stoichiometries for xylose use. The general applicability of this approach is outlined here, while as an outlook the detailed single strain optimization and the co-consumption of carbon sources *in silico* and *in vivo* should be worked on.

## Data Availability Statement

All datasets generated for this study are included in the article/Supplementary Material.

## Author Contributions

IB performed all molecular engineering and characterization experiments, analyzed the data, prepared figures, conducted the *in silico* experiments, and wrote the manuscript. TT provided guidance on *P. putida* biotechnology and edited the manuscript. TT, AW, and FR discussed the data and critically revised the manuscript. LB advised on all experiments, analyzed and discussed data, and edited the manuscript.

### Conflict of Interest

The authors declare that the research was conducted in the absence of any commercial or financial relationships that could be construed as a potential conflict of interest.

## Footnotes

^1^www.gurobi.com
