# Supplementary material for: Comparison of Three Xylose Pathways in Pseudomonas putida KT2440 for the Synthesis of Valuable Products
Source: Front Bioeng Biotechnol. 2020 Jan 17;7:480. doi: 10.3389/fbioe.2019.00480 (PMC6978631; doi:10.3389/fbioe.2019.00480)
Supplement: Supplementary file 1 [file Table_1.DOCX]

Supplementary Material

Comparison of three xylose pathways in *Pseudomonas putida* KT2440 for the synthesis of valuable products

Isabel Bator^1^, Andreas Wittgens^2,3,4^, Frank Rosenau^2,3,4^, Till Tiso^1^, Lars M. Blank^1,*^

^1^ iAMB - Institute of Applied Microbiology, ABBt – Aachen Biology and Biotechnology, RWTH Aachen University, Aachen, Germany

^2^ Institute for Pharmaceutical Biotechnology, Ulm-University, Ulm, Germany

^3^ Ulm Center for Peptide Pharmaceuticals, Ulm, Germany

^4^ Max-Planck-Institute for Polymer Research Mainz, Synthesis of Macromolecules, Mainz, Germany

*** Correspondence:** Lars M. Blank
lars.blank@rwth-aachen.de

# Supplementary Figures and Tables

## Supplementary Figures





**Supplementary Figure 1**: Consumed xylose and formed xylonate by xylose utilizing *P. putida* strains during production of mono-rhamnolipids or pyocyanin. (A) Consumed xylose at the end of the cultivation and (B) formed xylonate, which was a leftover at the end of the cultivations of rhamnolipid and phenazine production strains. Error bars indicate deviation from the mean (n=3).

## Supplementary Tables

Supplementary Table 1: List of oligonucleotides used in this work.

| Name | Direction | Used for | Sequence |
| --- | --- | --- | --- |
| 4_Pput_glmS  5_Pput_glmS  IB-5  IB-6  IB-27  IB-28  IB-29  IB-30  IB-31  IB-32  IB-33  IB-34  IB-35  IB-63  IB-64  IB-65  IB-66  IB-67  IB-68  IB-74  IB-87  IB-88  IB-89  IB-90  IB-91  IB-92  IB-93  IB-94  IB-118  IB-124  IB-125  SK06  SK07  SK08  SK09 | fwd  rev  fwd  rev  fwd  rev  fwd  rev  fwd  rev  fwd  rev  rev  fwd  rev  fwd  rev  rev  fwd  fwd  fwd  rev  fwd  rev  fwd  rev  rev  fwd  rev  rev  fwd  rev  fwd  fwd  rev | *P. putida* gDNA  *P. putida* gDNA  pBT-/pEMG-plasmids  pEMG-plasmids  *E. coli* gDNA  *E. coli* gDNA  *P. putida* gDNA  *P. putida* gDNA  *P. putida* gDNA  *P. putida* gDNA  *P. putida* gDNA  *P. putida* gDNA  pBT-plasmids  *P. taiwanensis* gDNA  *P. taiwanensis* gDNA  *P. taiwanensis* gDNA  *P. taiwanensis* gDNA  pBT-Weimberg  *P. taiwanensis* gDNA  pBT-Weimberg  *P. putida* gDNA  *P. putida* gDNA  *P. putida* gDNA  *P. putida* gDNA  *P. putida* gDNA  *P. putida* gDNA  pBT-Dahms  pBT-Dahms  *E. coli* gDNA  pBT-Weimberg  *E. coli* gDNA  pBG14ffg  pBG14ffg  *P. aeruginosa* gDNA  *P. aeruginosa* gDNA | AGTCAGAGTTACGGAATTGTAGG  GTCGAGAAAATTGCCGAGCT  CAAGGCGATTAAGTTGGG  TCCGGCTCGTATGTTGTG  caggtaccgaattcctcgagaggaggtgttcaaATGCAAGCCTATTTTGAC  ggcccgacgtcgcatgctccTTACGCCATTAATGGCAG  taacagggtaatctgaattcAGCGCCATGCCGTAGGCTTTG  agaacctacgGCGACACCGCTCCCGCAG  gcggtgtcgcCGTAGGTTCTCCGTCAGG  ttgcatgcctgcaggtcgacTTCCAACCTCGAATCCGG  GAGCACTTCCTGCAACTG  GGTGGCATCTGGTACAAC  ATACGCAAACCGCCTCTC  caggtaccgaattcctcgagaggaggtGGATCACATGTCGGACCTG  catggcatcaacctcctTCAGCGAATCGGTTCACG  gattcgctgaaggaggtTGATGCCATGCCGATAAC  ggcccgacgtcgcatgctccTCAATGAGAATGCCGTGG  ACCTCCTTCAGCGAATCG  accgattcgctgaaggaggtAATGACATGACCGATACC  GGAGCATGCGACGTCGGG  taacagggtaatctgaattcCCTACCTCAAGCCATACAC  agcgagccgtCTGTGTTCCCTTGCTTTG  gggaacacagACGGCTCGCTGCGTCAGG  ttgcatgcctgcaggtcgacTCGGCAGCGGTTTGCCTTC  GGTGTTTCCAGCCTGTTC  GTACTGCGGTGCTTGAAC  TCAATCCTCCGAAACGCGAC  AGGAGGTAATGACATGACCGATACC  ggcccgacgtcgcatgctccTCAGCAAAGCTTGAGCTG  acctcctTCAATGAGAATGCCGTGG  tgccacggcattctcattgaaggaggtGATCTCATGCCGCAGTCC  TAGAAAACCTCCTTAGCATG  GAATTCGAGCTCGGTACC  catgctaaggaggttttctaATGCGGCGCGAAAGTCTG  cgggtaccgagctcgaattcTCAGGACGCAGCCTTCAG |
